# Is 3-Tesla Gd-EOB-DTPA-Enhanced MRI with Diffusion-Weighted Imaging Superior to 64-Slice Contrast-Enhanced CT for the Diagnosis of Hepatocellular Carcinoma?

**DOI:** 10.1371/journal.pone.0111935

**Published:** 2014-11-06

**Authors:** Bettina Maiwald, Donald Lobsien, Thomas Kahn, Patrick Stumpp

**Affiliations:** Department of Diagnostic and Interventional Radiology, University of Leipzig, Leipzig, Germany; West German Cancer Center, Germany

## Abstract

**Objectives:**

To compare 64-slice contrast-enhanced computed tomography (CT) with 3-Tesla magnetic resonance imaging (MRI) using Gd-EOB-DTPA for the diagnosis of hepatocellular carcinoma (HCC) and evaluate the utility of diffusion-weighted imaging (DWI) in this setting.

**Methods:**

3-phase-liver-CT was performed in fifty patients (42 male, 8 female) with suspected or proven HCC. The patients were subjected to a 3-Tesla-MRI-examination with Gd-EOB-DTPA and diffusion weighted imaging (DWI) at b-values of 0, 50 and 400 s/mm^2^. The apparent diffusion coefficient (ADC)-value was determined for each lesion detected in DWI. The histopathological report after resection or biopsy of a lesion served as the gold standard, and a surrogate of follow-up or complementary imaging techniques in combination with clinical and paraclinical parameters was used in unresected lesions. Diagnostic accuracy, sensitivity, specificity, and positive and negative predictive values were evaluated for each technique.

**Results:**

MRI detected slightly more lesions that were considered suspicious for HCC per patient compared to CT (2.7 versus 2.3, respectively). ADC-measurements in HCC showed notably heterogeneous values with a median of 1.2±0.5×10^−3^ mm^2^/s (range from 0.07±0.1 to 3.0±0.1×10^−3^ mm^2^/s). MRI showed similar diagnostic accuracy, sensitivity, and positive and negative predictive values compared to CT (AUC 0.837, sensitivity 92%, PPV 80% and NPV 90% for MRI vs. AUC 0.798, sensitivity 85%, PPV 79% and NPV 82% for CT; not significant). Specificity was 75% for both techniques.

**Conclusions:**

Our study did not show a statistically significant difference in detection in detection of HCC between MRI and CT. Gd-EOB-DTPA-enhanced MRI tended to detect more lesions per patient compared to contrast-enhanced CT; therefore, we would recommend this modality as the first-choice imaging method for the detection of HCC and therapeutic decisions. However, contrast-enhanced CT was not inferior in our study, so that it can be a useful image modality for follow-up examinations.

## Introduction

Hepatocellular carcinoma (HCC) is one of the most common malignancies worldwide. Liver cirrhosis is a precancerous condition associated with the development of HCC. Other important risk factors include chronic hepatitis B and C, as well as alcohol abuse. The sequential carcinogenesis from regenerative nodules to overt HCC has been described previously, and the *de novo* development of HCC without prior liver cirrhosis has also been delineated [1]–[5].

HCC can be diagnosed by various imaging modalities, including ultrasound, multidetector computed tomography (CT) and magnetic resonance imaging (MRI). Despite these versatile imaging modalities, correct characterization of HCC versus other liver lesions remains a challenging task, and a definite diagnosis often cannot be made based on imaging alone [6]. However, in cancer patients, a precise diagnosis is important for optimal treatment planning [2], [7].

Concerning advanced magnetic resonance techniques, diffusion weighted imaging (DWI) has the ability to differentiate between malignant and benign liver lesions [8]–[12]. A recently developed liver-specific contrast medium, Gd-EOB-DTPA (Gadolinium-ethoxybenzyl-diethylene-triamine-pentaacetic-acid), is a paramagnetic contrast agent with properties of extracellular and hepatobiliary contrast media for use in MR imaging of the liver. This reagent allows for dynamic perfusion imaging and the evaluation of liver function. Gd-EOB-DTPA is taken up in hepatocytes to approximately 50% via OATP-1 (organic anion transporter protein-1), increasing the signal intensity of the liver parenchyma approximately 20 min after injection [13]–[16]. Several studies have demonstrated that this reagent improved the detection and characterization of focal liver lesions [17]–[19].

High field-strength MRI at 3.0 Tesla provides better tissue contrast compared to 1.5 Tesla due to a greater signal-to-noise ratio, improved image quality, higher resolution imaging and faster scanning times [20].

The aim of this study was to compare the diagnostic power of CT with 3 Tesla MRI using Gd-EOB-DTPA for the diagnosis of HCC and to evaluate the diagnostic impact of DWI with apparent diffusion coefficient (ADC) quantification in this setting.

## Material and Methods

### Patient Selection

Fifty patients (mean age 60.6 years, range 29–84 years, mean body weight 79,8 kg, range 45–120 kg, 42 male, 8 female, Table 1) with suspected or proven HCC were included in this prospective single-centre study to evaluate the diagnostic performance of contrast-enhanced CT and Gd-EOB-DTPA-enhanced MRI in terms of lesion detection. Inclusion criteria were suspicious findings in the US or/and increased laboratory parameters (e.g., alpha-fetoprotein). Exclusion criteria were renal failure, allergy to contrast agents, hyperthyreoidism, pregnancy and, especially for the MRI-examination, pacemaker or other non-compatible implants and claustrophobia. The aetiology of liver cirrhosis in the patient cohort was as follows: 26 patients with alcohol induced liver cirrhosis, 2 with Hepatitis B- and 3 with Hepatitis C-related chronic liver disease, 3 patients with hemochromatosis, one with Budd-Chiari-Syndrome and one with non-alcoholic steatohepatitis. 14 patients had cryptogenic liver cirrhosis. Based on the Child-Pugh-Classification, the severity of liver cirrhosis was classified as class A in 27 patients, class B in 16 patients and class C in 7 patients [Table 1].

**Table 1:** Demographics, aetiology of liver cirrhosis and patients clinical condition.

*Gender*	
Male	42
Female	8
***Mean age (years)***	60.6 (range 29–84)
***Mean weight (kg)***	79.8 (range 45–120)

*Aetiology of liver cirrhosis*	
Alcoholic disease	26
Hepatitis B	2
Hepatitis C	3
Haemochromatosis	3
Budd-Chiari-Syndrome	1
Non-alcoholic steatohepatosis	1
Cryptogenic	14

*Child Pugh Status*	
A (5–6)	27
B (7–9)	16
C (10–15)	7

The histopathological report after resection or biopsy of a lesion served as the gold standard for diagnosis, whereas a surrogate of follow-up (after 6 months) or complementary imaging technique (ultrasound, digital subtraction angiography) in combination with clinical (loss of weight, general state) and paraclinical parameters (especially alpha-fetoprotein) was used in unresected lesions.

This study was approved by the ethics committee of the medical faculty of the University of Leipzig, and all patients provided written informed consent.

### Imaging technique

Multiphase-CT was performed using two different scanners (Brilliance 64/iCT; Philips Healthcare, Eindhoven, Netherlands) with identical parameters to prevent bias within the CT: collimation of 0.625 mm, rotation time of 0.75 s, tube voltage of 120 kV, tube current 200 mAs and adjusted with automatic dose modulation, reconstructed slice thickness of 3 mm, matrix 512*512). The contrast agent (Iopromide Ultravist 370, Bayer Vital GmbH, Leverkusen, Germany) was applied at a constant volume of 100 ml at a rate of 3 ml/s (Power injector mississippi, Ulrich Medical, Ulm, Germany). The unenhanced phase, early arterial phase 10 s after bolustracking (positioning the respective region of interest in the abdominal aorta just above the coeliac trunk, threshold 150 HU) and portal venous phase 60 s after reaching the threshold were acquired.

Subsequent MRI (median time: 2.2 days, range 0–30d) was performed in all subjects using a 3.0 Tesla scanner (TrioTim, Siemens Medical Solutions, Erlangen, Germany). The study protocol consisted of the following sequences:

1. T2w-HASTE (half-fourier acquisition single-shot turbo spin echo) coronal and axial
2. T1w-VIBE (volume-interpolated breath-hold examination) coronal unenhanced, axial unenhanced and dynamic after contrast medium was applied (Gd-EOB-DTPA (Primovist), Bayer Vital GmbH, Leverkusen, Germany) at 0.1 ml/kg bodyweight at a rate of 2 ml/s using a power injector (Spectris solaris EP, Medrad, Dusseldorf, Germany), followed by a 30 ml saline flush. Scanning times were as follows: arterial phase, 2 s; portalvenous phase, 30–40, equilibrium phase, 2–3 min; and hepatobiliary phase, 20 min after the contrast bolus reached the abdominal aorta.
3. Diffusion-weighted sequence coronal and axial (b-value 0, 50 and 400 s/mm^2^)
4. T2w TSE (turbo spin echo) with fat saturation coronal
5. T1w in phase and opposed phase axial

See Table 2 for more details concerning the sequences.

**Table 2:** MR Imaging Parameters.

Parameter	T2-weighted (HASTE)	T1-weighted (VIBE)	DWI	T2-weighted TSE	T1-weighted in and out of phase
Imaging plane	Coronal and axial	Coronal and axial unenhanced, axial enhanced	Coronal and axial	Coronal	Axial
Fat saturation	No	Yes	Yes	Yes	No
Respiratory triggering	Breath-hold (12 s)	Breath-hold (16 s)	Respiratory-triggered	Respiratory-triggered	Breath-hold (18 s)
Repetition time (TR)	800 ms	2.92 ms	2000 ms	2000 ms	212 ms
Echo time (TE)	83 s	0.86 ms	60 ms	81 ms	2.32 ms
Flip angle	160°	10°	-	120°	65°
Bandwidth	781 Hz/Px	540 Hz/Px	1736 Hz/Px	260 Hz/Px	930 Hz/Px
Field of view (FOV)	450 mm	400 mm	380 mm	400 mm	380 mm
Slice thickness	5 mm	3 mm	5 mm	5 mm	5 mm
Matrix	320*256	256*200	192*154	320*224	256*200

### Imaging analysis

**Figure 1:**
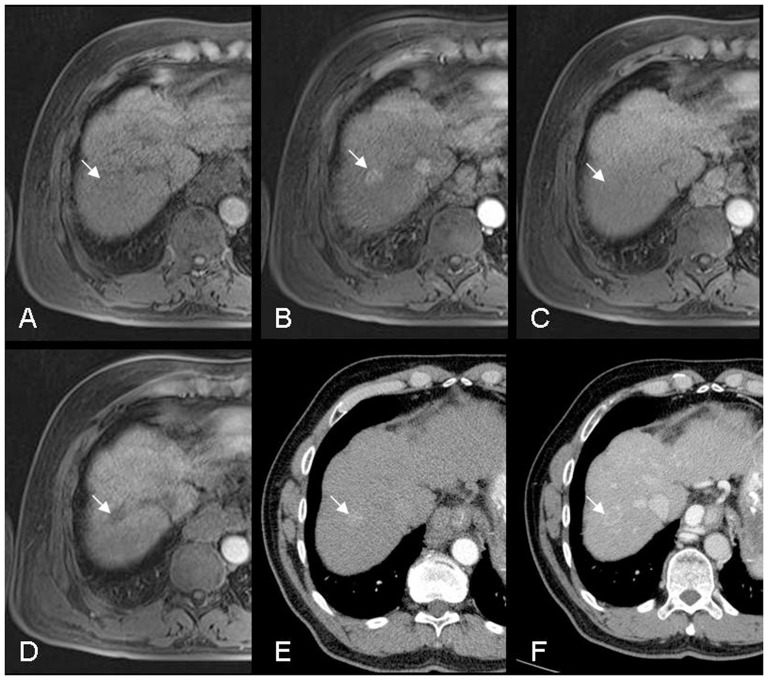
A 59-year-old male patient with liver cirrhosis (Child A) and HCC (arrow) in segment 7. Axial images: A) lesion is barely visible using unenhanced T1w-VIBE, B) marked arterial enhancement in T1w-VIBE following i.v. administration of contrast medium, C) typical washout of the lesion in the equilibrium phase (T1w-VIBE), and D) a clear hypointense lesion in the hepatobiliary phase (20 min after contrast agent injection, T1w-VIBE). Similar behaviour was observed with typical contrast medium enhancement in CT: E) early arterial phase after bolus tracking and F) washout in the portal venous phase with pseudocapsule.

Image analysis focused on the number, size and detectability of liver lesions, as well as image quality. A radiologist with 10 years of experience in abdominal imaging performed the analysis using a picture archiving and communication system workstation (MagicView 1000, Siemens Medical Solutions, Erlangen, Germany). The observer was aware of the patients being at risk of HCC, but otherwise blinded to all patient data. Diagnosis of HCC was based on hypervascularization in the arterial phase and washout in the portal venous phase or delayed phase, as suggested by the European Association for the Study of the Liver and the American Association for the Study of Liver Disease for MRI and CT [1]. In addition, focal areas with a suspicious hypointense signal in the hepatobiliary phase were used to detect HCC [21] (Figure 1). The radiologist recorded the presence and anatomical location of lesions, as well as diagnostic confidence using the following 5-point scale: 1 =  definitely not HCC, 2 =  probably not HCC, 3 =  equivocal, 4 =  probably HCC, 5 =  definitely HCC.

Lesion detectability and image quality were evaluated using a 5-point rating-scale (1 =  excellent, 2 =  good, 3 =  fair, 4 =  poor, 5 =  unacceptable). The average largest tumour diameter was determined using a measuring tool integrated in the workstation software.

ADC-values were measured for each clearly demarcated lesion in ADC-map by drawing a circular region of interest into the tumour that encompassed as much of the lesion as possible while excluding vascular structures and necrotic tissue.

Imaging analysis was accomplished according to the following settings:

1) In general, CT-scans were compared with

2) complete MRI-examinations (including conventional dynamic MRI with hepatobiliary phase and DWI).

To estimate the impact of the hepatobiliary phase and diffusion-weighted imaging, MRI-data were subdivided into three sets:

3) conventional dynamic MRI without the hepatobiliary phase and DWI

4) dynamic MRI including the hepatobiliary phase

5) MRI including diffusion-weighted sequences.

In addition, the observer evaluated the reading time.

### Statistical analysis

Statistical analysis was performed using SPSS 20.0 for Windows (statistical package for social sciences 20.0, Chicago, IL, USA) and Microsoft Excel (Microsoft, Redmond, WA, USA). The diagnostic performance of each technique was assessed by measuring the area under the curve (AUC) of the free-response receiver operating characteristic analysis (ROC-curve) on a lesion-per-patient-basis.

Sensitivity, specificity, and positive and negative predictive values were calculated for patients assigned a diagnostic confidence level of 4 and 5 (probably and definitely HCC). In addition, we included patients with a confidence level of 3, because in clinical routine, a suspicious lesion must be clarified (e.g., by further imaging or biopsy). The differences in the ROC-curves, sensitivities, specificities, positive predictive value (PPV) and negative predictive value (NPV) were statistically analysed using a binomial test. Student's t-test was used to calculate significant differences for image quality, detectability and reading time between the image modalities, P-values <0.05 were considered significantly different.

## Results

In 35 of 50 patients, the histopathological report after resection or biopsy of a lesion served as the gold standard for diagnosis, and in 24 of these 35 patients, the diagnosis of HCC was proven histopathologically. In 2 additional cases, HCC was diagnosed at follow-up (after 6 months) via clinical and paraclinical parameters.

In our study, 26 of 50 patients were positive for HCC (MRI-related: 9 patients with one lesion, 4 patients with 2 lesions, 3 patients with 3 lesions and 10 patients with 4 or more lesions).

**Figure 2:**
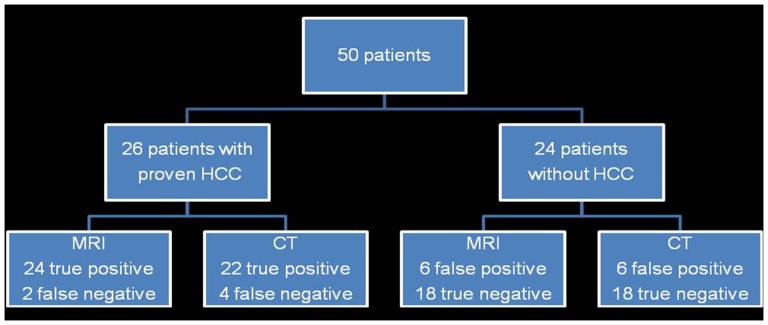
Flow chart for identification of patients with HCC in MRI and CT.

ROC-curves for MRI displayed similar AUCs as observed for CT (0.837 vs. 0.798, p = 0.48). Sensitivity and positive and negative predictive values were measured for both methods (sensitivity 92%, PPV 80% and NPV 90% for MRI vs. sensitivity 85%, PPV 79% and NPV 82% for CT). Specificity was 75% for both techniques (see Table 3 and Figure 2). False positives resulted from numerous metastases of a neuroendocrine carcinoma, one adenoma and regenerative nodules. Because we calculated sensitivity, specificity, and positive and negative predictive values on a per-patient-basis, our subset analyses revealed no differences.

**Table 3:** Diagnostic accuracy, sensitivity, specificity, PPV and NPV for MRI and CT (binomial test, p<0.05 =  significant).

	MRI	CT	P-value
**Diagnostic accuracy (AUC)**	0.837	0.798	0.4795
**Sensitivity**	0.92	0.85	0.4795
**Specificity**	0.75	0.75	1.000
**PPV**	0.80	0.79	0.500
**NPV**	0.90	0.82	1.000

**Figure 3:**
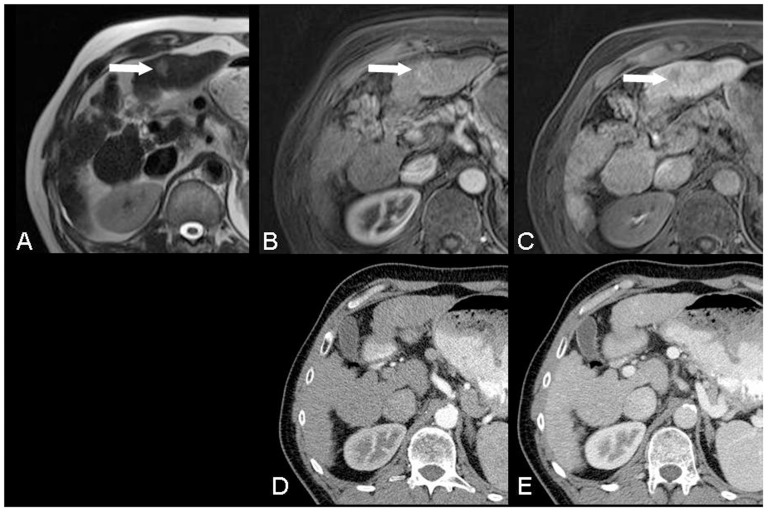
A 59-year-old male patient with liver cirrhosis and HCC (arrow) in S3 was only observed using MRI: *A*) markedly hyperintense HCC in T2w-HASTE axial, *B*) typical arterial enhancement in T1w-VIBE, and *C*) hypointense lesion in the hepatobiliary phase. No lesion was detected using CT: *D*) early arterial phase and *E*) portal venous phase.

**Figure 4:**
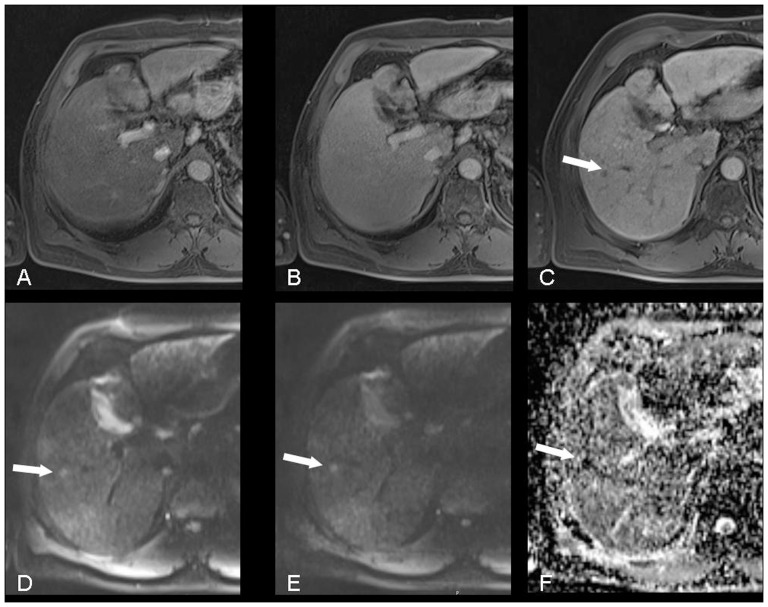
MRI of a 53-years-old male patient with HCC (arrow) in Segment 5: no lesion was identified in the arterial (A) and equilibrium phases (B), a small hypointense lesion was only observed in the hepatobiliary phase (C) and in DWI, where it is seen as a hyperintense lesion in b 50- (D) and b 400-images (E) and hypointense in the ADC-map (F).

On a *liver-lesion-per-patient basis*, we detected slightly more lesions that were suspicious for HCC using MRI compared to CT, but this value did not reach statistical significance (mean, 2.7 for MRI versus 2.3 for CT, p = 0.256, Figure 3). One additional HCC was identified with aid of the hepatobiliary phase compared to conventional dynamic MRI; this lesion measured 8 mm in diameter (Figure 4). Compared to Gadolinium-EOB-DTPA-enhanced MRI-images evaluated with the hepatobiliary phase, no additional lesions were detected by DWI. However, DWI identified one additional malignant lesion compared to conventional dynamic MRI-scan. This was the same 8 mm diameter lesion that was observed with the hepatobiliary phase, and this finding impacted patient treatment (Figure 4).

*Lesion size* was similar using both methods, with an average greatest diameter of 33 mm for CT and 32 mm for MRI (measured in the arterial phase T1w VIBE; p = 0.195). Twenty malignant neoplasms were >3 cm, 16 lesions were 2–3 cm and 35 lesions were <2 cm, as determined by MRI-scans.

**Figure 5:**
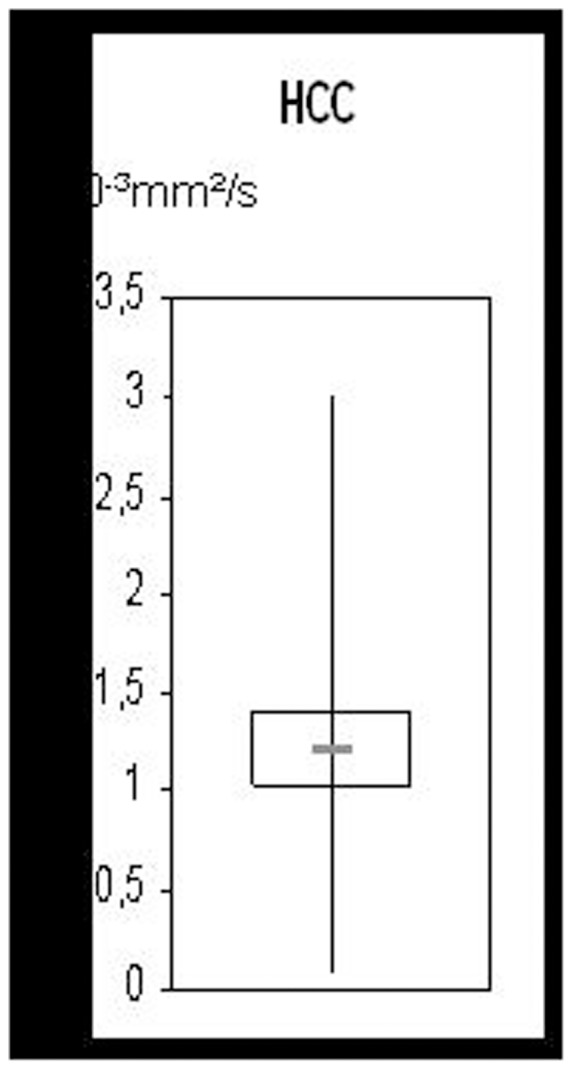
Range of ADC-values (10^−3^ mm^2^/s) in lesions suspicious for HCC.

*ADC-measurements* in 32 lesions showed extremely heterogeneous values, with a mean of 1.2±0.5×10^−3^ mm^2^/s (range, 0.07±0.1 to 3.0±0.1×10^−3^ mm^2^/s; Figure 5).

The radiologist reported that the *detectability* of lesions was similar using both methods using a 5-point rating scale (2.6 for CT and 2.7 for MRI, p = 0.807; range from 2.6 to 4.0 between sequences, 1 =  excellent, 5 =  unacceptable). The ability of MRI to detect lesions was significantly better using the hepatobilary phase (2.2, range from 1 to 4) compared to conventional dynamic MRI (2.9, p = 0.005), but the difference was not significantly different compared to DWI (2.6, p = 0.125).

*Image quality* (2.2 for CT vs. 2.3 for MRI, p = 0.249, 2 =  good, 3 =  fair) was more scattered within MR sequences (1.8 in T1w HASTE and 2.9 in T2w TSE). Image quality was rated better for the hepatobiliary phase and conventional dynamic MRI than for DWI (2.0 for hepatobiliary phase, 2.1 for dynamic MRI without hepatobiliary phase and 2.7 for DWI, p<0.001). No significant disparity was noted between conventional dynamic MRI and the hepatobiliary phase (p = 0.342).

The average *reading time* for MR-images was16.9 min and significantly longer than the reading time for CT-scans, which averaged 4.5 min. (p<0.001).

## Discussion

### Accuracy

In our study, MRI showed similar diagnostic accuracy for the detection of HCC compared to CT. Reports by Akai et al. and Lee et al. demonstrated similar results with a tendency to higher diagnostic accuracy for MRI, but also without statistically significant differences [22], [23]. One explanation for this discrepancy might be the high number of suspicious lesions with a diameter greater than 3 cm in our study population. Haradome et al. also showed no difference in diagnostic accuracy between conventional dynamic MRI and CT. However, using the hepatobiliary phase, MRI displayed significantly higher accuracy than CT, especially for lesions smaller than 1.5 cm [24]. Kim demonstrated that MRI has better sensitivity for the detection of HCCs due to an increased delineation of hypointensity of HCC at a three-minute late phase and a hepatocyte phase [25], supporting previous reports [26]–[28]. While these studies report that CT is inferior to MRI, the use of different contrast agents, older scanner technology and different scanning parameters of the CTs must be taken into account. Similar to our study parameters, several groups used an early arterial phase [25], [27]. For example, Chan et al. described better conspicuity for hepatocellular carcinomas using a bolus tracking delay of 6s for achieving the arterial phase [29]. Other studies state that the late arterial phase (e.g., approximately 14–30 s from 100 HU-threshold) is the optimal scan window for the detection of HCC [30]. Moreover, differences in histopathological subtypes of HCC might also yield different enhancement patterns [31], making it difficult to clearly specify standard examination protocols.

### Detectability and number of lesions

Although the reader rated the subjective detectability of liver lesions similarly for MRI and CT, slightly more liver lesions per patient were detected using 3T MRI. Although this did not reach statistical significance, it is important for therapeutic decisions because liver transplantation can achieve excellent results in patients with HCC according to the benchmark defined by the Milan criteria (solitary HCC of less than 5 cm or with up to three nodules of less than 3 cm) [32]–[34]. Furthermore, decision to surgically resect tumours or use minimally invasive therapies (i.e., radiofrequency ablation (RFA), laser-induced interstitial thermotherapy (LITT), microwave ablation (MWA), cryoablation and transarterial chemoembolisation (TACE), etc.) depends on number and size of hepatocellular carcinomas, as visualised using CT or MRI [35], [36].

In our study, the detectability of lesions using MRI with the hepatobiliary phase 20 min after i.v. injection of Gd-EOB-DTPA was significantly better than conventional dynamic MRI. In one patient a suspicious lesion with a diameter of 8 mm was only observed with the hepatobiliary phase and DWI, but not with conventional dynamic MRI (Figure 4). This lesion changed the therapeutic management of the patient because it was the 4^th^ HCC suspicious lesion in his liver, which excluded him from the liver transplantation list according to the Milan criteria [32]. These data support several studies reporting that Gd-EOB-DTPA enhanced MRI is superior to conventional dynamic MRI [17], [21], [24].

Gd-EOB-DTPA is a gadolinium-based, liver specific MRI contrast medium that allows diagnosis derived from haemodynamics during the extracellular phase and measures hepatocellular function during the hepatobiliary phase. Information regarding the degree of cellular differentiation might also be possible [18]. Concerning the timing of hepatobiliary phase imaging, Motosugi et al. described that if the liver parenchyma is sufficiently enhanced 10 min after injection, no further imaging is necessary to detect focal liver lesions. However, the visual liver to spleen contrast scores 20 min after injection were frequently higher than 10 min images in patients with chronic liver diseases. These data indicate that a longer delay of 20 min might be more useful for patients with chronic liver diseases [37]. Because all of our patients suffered from chronic liver disease, we acquired hepatobiliary phase images 20 min after contrast application.

### DWI

DWI is commonly used in liver imaging to assess various focal lesions. In particular, DWI has a higher detection rate and diagnostic performance for small, malignant liver lesions compared to conventional dynamic MRI with different contrast agents; however, these results are not always significant [38]–[40]. A recent study by Holzapfel et al. reported no significant difference in diagnostic accuracy and sensitivity between diffusion weighted imaging, Gd-EOB-DTPA-enhanced imaging and combined imaging for the detection of focal liver lesions. However, for lesions smaller than 10 mm, a combination of DWI and Gd-EOB-DTPA significantly increased the overall detection rate. Similar to our findings regarding HCC-related diagnostic accuracy, Gadolinium-enhanced MRI and the combination of DWI and Gd-EOB-DTPA enhanced MRI demonstrated equal results [41]. In our 50 patients, just one additional HCC suspicious lesion was detected with DWI compared to conventional dynamic MRI, an 8 mm lesion that was also detected with hepatobiliary phase imaging. Therefore, DWI did not improve the detection of HCC compared to imaging with Gd-EOB-DTPA in our study.

Park et al. also demonstrated that DWI was outperformed by contrast enhanced T1-weighted imaging for the detection of HCC, but it represents a reasonable alternative [42]. However, if Gd-EOB-DTPA is used in the hepatic imaging, a time gap occurs between the equilibrium phase and the hepatobiliary phase, and because there is no significant impact of contrast media on achieving diffusion-weighted imaging and ADC-maps [43]–[45]. This gap can easily be filled with respiratory-triggered, diffusion-weighted imaging, which can provide additional information for the characterization of focal liver lesions. An important advantage of DWI is that no contrast agent is necessary, a property that is especially valuable for patients with poor renal function [39]. In our department, diffusion-weighted MR imaging is part of the routine liver protocol for all patients.

The potential to differentiate between benign and malignant liver lesions using ADC-quantification was previously reported in the literature [8]–[12]. Several thresholds have been proposed to accomplish this task, but there is still considerable overlap between benign and malignant liver lesions [9]. Vandecavaeye confirmed that there is no significant difference in ADC between malignant and benign lesions in patients with cirrhotic liver disease [46]. In our study, the mean ADC-value of HCC lesions was 1.2±0.5×10^−3^ mm^2^/s, which is similar to values reported in the literature. For example, Naoto measured an ADC-value of 1.31±0.28×10^−3^ mm^2^/s [12], and Holzapfel reported an ADC-value of 1.12±0.28×10^−3^ mm^2^/s for a small number of HCC samples [9]. These variances are likely due to differential cellularity of the tumours [47]. Another influential factor are the b-values chosen for DWI. In our study, we used relatively low b-values (b = 0, 50 and 400 s/mm^2^), which have the disadvantage of being influenced by perfusion effects. Measured ADC-values tend to decrease as the b-value increases [48]. However, the use of low (perfusion-sensitive) b-values has several advantages: it provides a higher signal-to-noise ratio and more anatomical information of the liver, it is less sensitive to eddy current-induced distortions and it suppresses signals from the hepatic vasculature, which improves the detectability of perivascular lesions [49]–[51]. Further investigations are needed to determine the best b-values for liver imaging.

Our study has some limitations. First, there was a bias in patient recruitment because we included patients with proven or suspected HCC. This stipulation might result in an overestimation of specificity. Second, we could not achieve a histological proof for every detected lesion due to ethical reasons, so we had to use follow-up examinations and surrogates of clinical and paraclinical findings to confirm the presence of lesions. For this reason, we calculated sensitivity, specificity, and positive and negative predictive values on a patient basis. Third, our study group was relatively small, and further studies with more patients might yield statistically significant results. Fourth, we used a first generation MR-scanner with 3 Tesla, which can suffer from B0 artefacts within the liver parenchyma. Newer MR-Scanners with different coil and RF impulse designs (i.e., the TrueForm and MultiTransmit) reduce these artefacts and can increase the diagnostic ability for liver imaging. Finally, we only determined ADC-values for suspicious lesions that were clearly visible in the ADC-map. However, our ADC-values were comparable to the values given in literature.

## Conclusions

Our study did not show a statistically significant difference in detection of HCC between 3-Tesla Gd-EOB-DTPA-enhanced MRI with diffusion-weighted imaging and 64-slice contrast-enhanced CT.

As we detected slightly more lesions per patient using MRI, we recommend this imaging modality as the first-choice imaging method for the detection of HCC and individual therapeutic decisions. However, contrast-enhanced CT was not inferior in our study, indicating that it represents a useful image modality when MRI is not available or for follow-up examinations.
